# Exploring the effect of Jasmonic Acid for Aphids control for improving the yield of *Triticum aestivum* varieties

**DOI:** 10.7717/peerj.14018

**Published:** 2022-10-27

**Authors:** Huma Aslam, Sajida Mushtaq, Sadia Maalik, Naheed Bano, Emad M. Eed, Amir Bibi, Ayesha Tahir, Iram Ijaz, Samina Tanwir, Amany S. Khalifa

**Affiliations:** 1 Department of Botany, University of Agriculture Faisalabad, Faisalabad, Pakistan; 2 Department of Zoology, Government College Women University Sialkot, Sialkot, Pakistan; 3 Department of Fisheries and Aquaculture, Muhammad Nawaz Sharif University of Agriculture, Multan, Pakistan; 4 Department of Clinical Laboratory Sciences, College of Applied Medical Sciences, Taif University, Taif, Saudi Arabia; 5 Department of Plant Breeding and Genetics, University of Agriculture, Faisalabad, Faisalabad, Pakistan; 6 Department of Biosciences, COMSATS University Islamabad, Islamabad, Pakistan; 7 Department of clinical Pathology and Pharmaceutics, College of Pharmacy, Taif University, Taif, Saudi Arabia

**Keywords:** Jasmonic acid, Aphids, Wheat, Antioxidants, Control

## Abstract

Many biotic and abiotic factors influence the production of wheat (*Triticum aestivum* L.). Among biological agents, aphids are destructive pests effecting wheat yield drastically. This study was designed to evaluate the impact of foliar Jasmonic acid spray on aphid population as well as on plant growth during aphid infestation in two wheat varieties *i.e.*, Borlaug-2015 and Zincol-2015. Plants are cultivated in pots and treated with jasmonic acid at concentrations of 0.1 and 1 mM (JA). The results revealed that length of shoot and roots decreased after aphid stress and was improved (21–24%) by JA spray. Photosynthetic pigments increased after applying the jasmonic acid spray compared to control plants. Jasmonic acid spray helped the plants to recover from aphid stress by enhanced production of antioxidant enzymes CAT (Catalase) (65–71%), SOD (Superoxide dismutase) (71–74%) and POD (Peroxidase) (61–65%). Consequent to improved defence system, plants treated with JA had fewer aphids as compared to control (60–73% reduction), 24 h after spray. The higher concentration of JA (1 mM) proved more effective as compared to 0.1 mM jasmonic acid. Moreover, Zincol-2015 appeared tolerant as compared to Borlaug-2015 against aphid infestation. The application of jasmonic acid as an exogenous foliar application showed an overall positive impact on the physiological and biochemical attributes of both varieties. It helps the plants to enhance resistance against the biotic stress and can be adopted as future alternative for aphid management. However, detailed studies regarding understanding of underlying molecular mechanisms are needed to optimize the mode for field application.

## Introduction

Wheat plays a crucial role in the economic stability of a country and is among the major cultivated crops around the world (Khan et al., 2011; Azam & Shafique, 2017). Wheat is the best source of many trace elements, dietary fibers, vitamins, and phenolic acids (Cornell & Hoveling, 2020). Whole grain is a crucial part of healthy food and helps lower the rate of chronic and acute diseases minimizing the risk of cardiovascular issues and diabetes (Bird & Regina, 2018). Pakistan is suffering from many problems regarding wheat production, such as low yield, old farming methods, short resources and lack of key inputs (Iqbal et al., 2005; Rehman et al., 2015).

Insects are important living animals which are considered eminent in many ecological ecosystem services (Rana et al., 2019; Maqsood et al., 2020; Ramzan et al., 2021). These are influenced by the different biotic and abiotic factors (Majeed et al., 2020; Majeed, Khawaja & Rana, 2021; Asghar et al., 2022; Majeed et al., 2022). Cereal aphids are a prominent wheat insect pest that tremendously impacts wheat productivity (Liu et al., 2020a). Aphids are an increasing threat to cereal crops, particularly wheat, and are one of the major reasons for low yield compared to neighboring countries. Aphids harm wheat plants directly and indirectly (Gong et al., 2021). Aphids can give rise to 35–40% direct loss of yield by sucking the sap of plants and an indirect loss of 20–80% by conveying fungal diseases and viruses (Khan et al., 2011). A vast range of plant species nourishes aphids. In many crops, aphids facilitate the pathogen and the transmission of viruses (Lu & Gao, 2016; Gong et al., 2021). In addition to yield, grain quality is compromised due to loss of valuable nutritional constituents and dough mixing properties are negatively affected (Kalaisekar, Padmaja & Patil, 2019). Aphids are diverse, and their association is very specific for the host and host stages of development. Wheat development is divided into germination, emergence, tillering and double ridges, terminal spikelet, beginning of stem elongation, anthesis, spike emergence and maturity (Papi, Ahmadi & Rafiei, 2016). Similarly, grain development is encompassed by growth and differentiation, milk stage, grain filling, dough stage and maturity (Tasleem-Tahir et al., 2012). In wheat, aphids attack at the early stages of development from seedling emergence but mainly peak at flowering and grain development stages and decline towards grain maturity (Ahmad & Nasir, 2001). Sometimes the aphid attack starts at the stem elongation stage and expanded along with the plants’ growth (Yahya et al., 2017).

Among various pest management strategies, jasmonic acid applications are effective against aphids and pest populations. Jasmonic acid increases plant resistance against aphid attacks and stimulates many physiological processes to recover from wounds caused by aphid attack (Cao, Wang & Liu, 2014). Jasmonic acid is considered a stress hormone that modulates the plant’s reaction against different biotic and abiotic stresses as it can regulate various metabolites such as terpenoids and phytoalexins (Gladman et al., 2019; Gomi, 2020). Externally applied jasmonic also positively influences plant growth as it enhances the growth of primary roots, length of plants and grain weight by regulating various phytohormones that are key regulators in the development of plant and stress response (Huang et al., 2017; Wang et al., 2020).

Wheat pest management involves regular monitoring and combining cultural techniques and biological treatments for long-term success (El-Wakeil & Volkmar, 2012; Hazafa et al., 2022). Alternative aphid protection measures must be developed (Richter, 2011; Liu et al., 2020b). In this study, we have investigated the impact of foliar Jasmonic acid spray on aphid populations and the influence of Jasmonic acid on the development of wheat plants during aphid infestation in two different wheat varieties: Borlaug-2015 and Zincol-2015. We also looked at how varying concentrations of jasmonic acid affected the development of wheat and the changes in associated enzymes and plant attributes.

## Materials and Methods

The pot experiment was carried out in a warehouse at the Entomology Department, University of Agriculture Faisalabad, Pakistan. The experiment was performed using garden soil in a completely randomized design. Seeds of two wheat varieties, *i.e.*, Borlaug-2015 and Zincol-2015, were collected from the Plant Breeding and Genetics Wheat Lab, University of Agriculture Faisalabad.

### Experimental treatment

The control plants were kept in a net to prevent aphids attack. Two dilutions of jasmonic acid (0.1 and 1 mM) were used, each with three replications. The treatments included control (no aphid, no JA spray) T0, no aphids plus JA spray (0.1 mM) T1, no aphid plus JA spray (1 mM) T2, aphid (no JA spray) T3, aphid plus JA spray (0.1 mM) T4 and aphid plus JA spray (1 mM) T5. After a natural aphid attack, T1, T4 and T2, T5 were sprayed with respective dilutions of jasmonic acid. The number of aphids was counted before and after 1, 3, and 7 days of spray. Harvest was taken after 1 week of the spray.

### Measurements of plant attributes

After harvest, all plants were rinsed with distilled water. At the experimental site, fresh weights were assessed instantly after harvesting using a digital weighing balance and after the plants had dried completely, the dry weight was measured. The total length of plant and root length were calculated by measuring tape. Fresh plant samples were kept at −30 °C in a biomedical freezer and proceeded for further analysis.

### Leaf relative water content (RWC)

RWC was evaluated using a Weatherley-established protocol (Weatherley, 1950). Briefly, leaf fresh weight was assessed after harvest. The leaves were soaked in distilled water to obtain turgid weight for 24 h and were subsequently dried at 70 °C till complete drying.

Leaf relative water content was estimated by this formula:

$Leaf\; relative\; water\; content\; \left( \% \right)=\displaystyle{{Leaf\; fresh\; weight. - \; Leaf\; dry\; weight.} \over {Leaf\; turgid\; weight\; - \; Leaf\; dry\; weight}} \times 100$

### Determination of photosynthetic pigments

Chlorophyll a, chlorophyll b, total Chlorophyll, and carotenoid concentrations were determined using the Arnon protocol (Arnon, 1949). After harvesting, fresh leaf samples measuring 0.1 g were obtained. These samples were dipped in 95% of acetone (8 ml) in dark at 4 °C for 24 h. Absorbance was recorded at 646-nm, 663-nm, and 450-nm to calculate Chlorophyll a, chlorophyll b, carotenoid and total chlorophyll, according to respective formulas, using a spectrophotometer.

### Antioxidant enzymes

For enzyme extraction, 0.5 g samples of the fresh plant were ground and homogenized in 3 mL phosphate buffer (50 mM) on ice. Then the volume was raised to 5 mL. After that, the samples were centrifuged for 15 min at 4 °C at a speed of 15,000 rpm. Before being used for enzymatic assays, the supernatant was separated, wrapped with aluminum foil, and stored at 4 °C.

### Superoxide dismutase (SOD)

SOD (EC# 1.15.1.1) activity was determined by using the protocol of Beyer & Fridovich (1987). The mixture of the reaction included NBT (50 µM), L-methionine (12 mM), (50 mM) sodium carbonate, (0.1 mM) EDTA, (10 µM) riboflavin, (50 mM) phosphate buffer having pH 7.6, as well as enzyme extract 100 µL, in the 3 mL final volume. The control reaction’s composition was the same, excluding crude extract. At room temperature, the reaction mixture in test tubes was exposed to fluorescent lighting for 15 min. After 15 min, the spectrophotometer measured the reading at 560 nm.

### Peroxidase (POD)

The Gorin & Heidema (1976) technique was used to assay the activity of POD (EC# 1.11.1.x). In this protocol, 4-methyl catechol was used as a substrate. It induced oxidation after combining with H_2_O_2_. In a total volume of 3 mL, the reaction mixture for POD measurement contains 5 mM 4-methyl catechol, 100 mM buffer of sodium phosphate (Na3PO4), 5 mM H_2_O_2_ and an extract of the enzyme 500 µL. A spectrophotometer was used to detect absorbance at 420 nm.

### Catalase (CAT)

The activity of CAT (EC# 1.11.1.6) was measured using Kumar’s technique (Kumar et al., 2010). In the reaction mixture used to determine the CAT, there was 2.8 mL of phosphate buffer (50 mM), 0.1 mL of H2O2 (300 mM), and 0.1 mL of the enzyme extract at the end. The test mixture’s absorbance at 240 nm was determined after a 30 s delay.

### Total soluble protein (TSP)

The Lowry et al. (1951) protocol was used to identify the TSP. To calculate the TSP, frozen plant leaves were grounded in phosphate buffer having a pH of 7.0 and blended on ice. The supernatant was separated after centrifuging the extract. The supernatant of about 0.1 ml was taken and diluted by raising the volume up to 1 ml. After that, alkaline copper sulfate of the same volume was added to these samples. The samples were vortexed for 10 min. Then, folin’s reagent was mixed into it. After that, the samples were incubated for 30 min at 28 ± 2 °C. A spectrophotometer was used to determine the absorbance at 650 nm.

### Phenolic content

The phenolic content was determined using the technique of Julkunen-Tiitto (1985). The crude extract, 50 µL was diluted using 2 mL of water. The extract was then treated with 1.0 mL of folin-ciocalteu’s phenol reagent. The mixture was vortexed for 2 min. Then immediately added 5 mL of Na_2_CO_3_ solution (20% concentration) and increased the volume to 10 mL. The mixture was shaken very well and thoroughly. After 10 min, the absorbance of samples for phenolic analysis was determined using a spectrophotometer at 700 and 735 nm.

### Malondialdehyde (MDA) content for lipid peroxidation estimation

Heath & Packer’s (1968) technique was used to evaluate malondialdehyde content. Briefly, fresh leaf samples of 0.1 g were taken. These samples were ground in a 0.1% TCA solution. After blending, the extract was transferred into the eppendorf tubes and centrifuged at 12,000 rpm speed in a centrifuged machine for 5 min. The supernatant (0.3 ml) was then incubated for 30 min at 95 °C with 1.2 ml of TBA (0.5%) in TCA (20%). A chilling ice batch was used to stop the reaction and after centrifugation (12,000 rpm, 10 min), supernatant was estimated at 532 nm and 600 nm.

### H_2_O_2_ content

A total of 500 mg of leaf samples were crushed thoroughly in 5 ml of 0.5% TCA. After being ground, the extract was centrifuged for 15 min at 12,000 rpm. In 1 ml of supernatant, 0.5 ml of potassium phosphate buffer was added. After that 1 ml of potassium iodide added in it. A spectrophotometer was used to evaluate the supernatant’s absorbance at 390 nm. A standard curve displayed the hydrogen peroxide (H_2_O_2_) content (Velikova, Yordanov & Edreva, 2000).

### Direct count of aphids

A direct count of aphids was done. The total number of aphids was estimated in each pot by counting aphids on plant leaves and tillers. The number of aphids was counted before spray, as well as on the 1st, 3rd, and 7th days after spray.

### Statistical analysis

The data under the CRD design were analyzed using ANOVA. The data was analyzed using Statistics 8.1 software. The LSD test with a probability level of 5.0 percent was used to compare treatment means.

## Results

### Plant attributes

**Figure 1:**
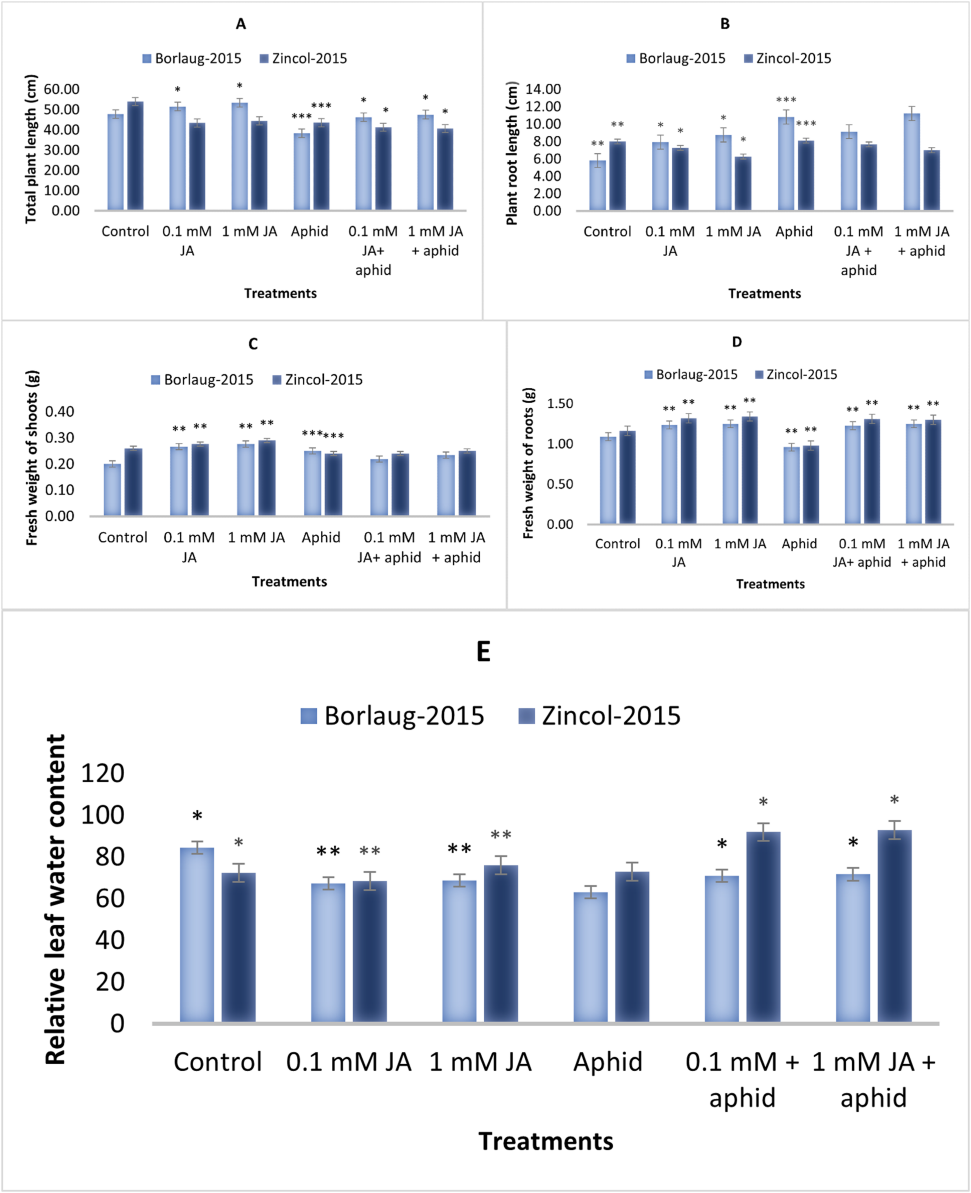
Effect of foliar application of jasmonic acid with and without aphid stress, on plant height (cm) (A), plant root length (cm) (B), shoot fresh weight (g) (C), root fresh weight (g) (D), and Leaf relative water content (E), of two varieties of wheat *i.e*., Borlaug-20. Values are per gram fresh weight of sample. Mean values ± SE of three replicates are presented. *p ≤ 0.05; **p ≤ 0.01; ***p ≤ 0.001.

The results showed that control plants had greater plant lengths than aphid-stressed plants. The jasmonic acid application significantly increased the plant height during aphid infestation. Aphid-stressed plants showed 38.3 and 43.5 cm of total plant length in Borlaug-2015 and Zincol-2015, respectively. After JA application, plant length improved (46–47 cm) in Borlaug-2015. In the present study, control plants had smaller root lengths than aphid-stressed plants. Greater plant root length was observed in 1 mM JA sprayed plants compared to 0.1 mM JA. Moreover, it was observed that aphid infestation reduced the fresh weight of the shoot. In aphid-stressed plants, fresh root weight was 0.96–0.98 g, which was increased after JA treatment to 1.25–1.30 g. This root and shoot weight reduction after aphid infestation was higher in Zincol-15 than in Borlaug-2015. The JA spray plants had more leaf relative water content (LRWC) than the aphid-stressed plants. A significant increase in LRWC was observed in JA spray plants (Fig. 1).

### Photosynthetic pigments

**Figure 2:**
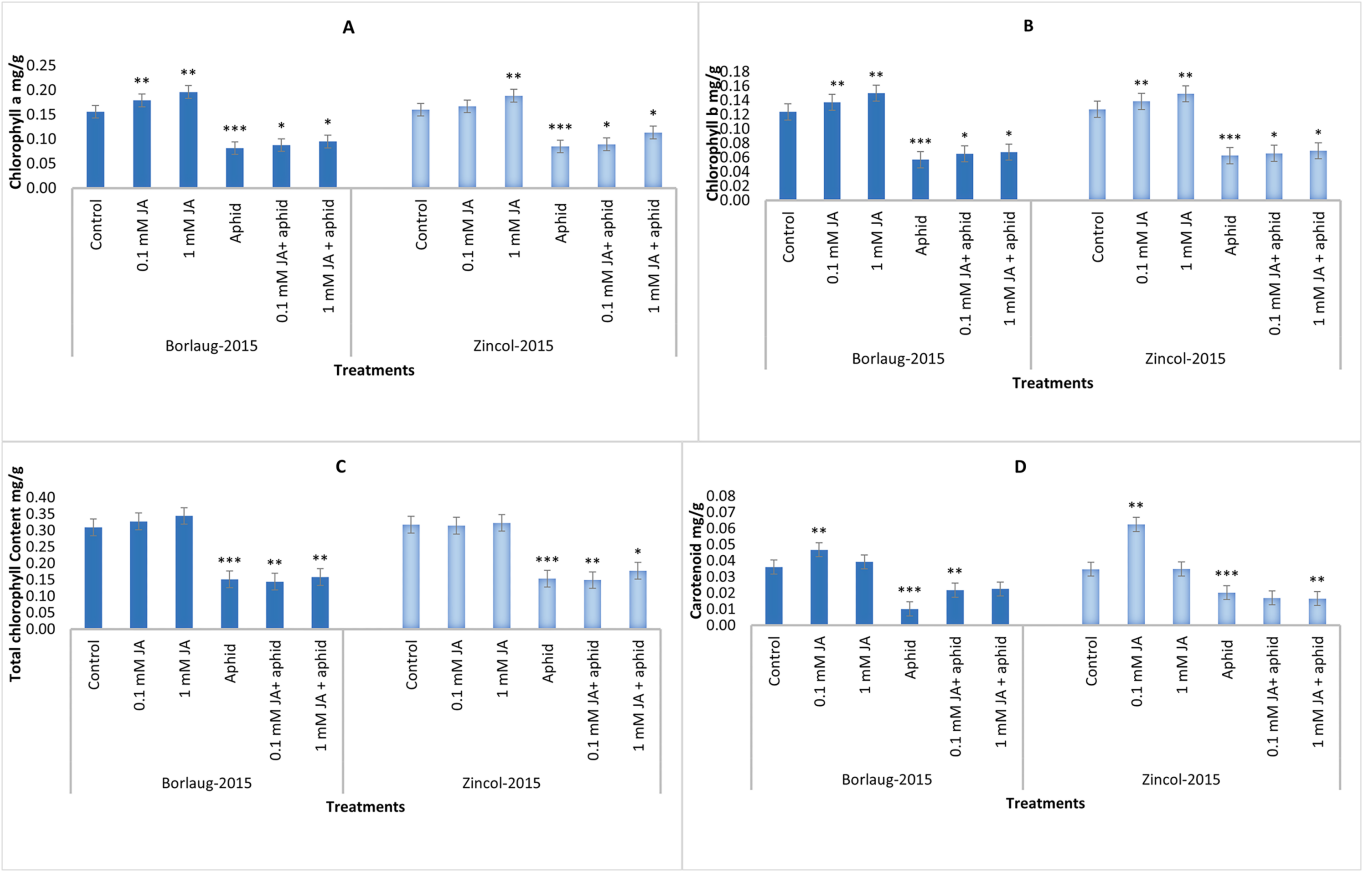
Impact of foliar jasmonic acid treatment with and without aphid stress, on Chlorophyll a (A), Chlorophyll b (B), total chlorophyll content (C), and Carotenoids content (D) of two varieties of wheat *i.e*., Borlaug-2015 and Zincol-2015. Values are per gram fresh weight of sample. Mean values ± SE of three replicates are presented. *p ≤ 0.05; **p ≤ 0.01; ***p ≤ 0.001.

The results revealed that aphid stress significantly reduced all photosynthetic pigments. Jasmonic acid spray significantly increased the content of Chlorophyll a, chlorophyll b, total Chlorophyll and carotenoid. The amount of Chlorophyll a enhanced from 0.08 to 0.09 mg/g f.wt in Borlaug-2015 and 0.08 to 0.11 mg/g f.wt in Zincol-2015 after JA application in aphid-stressed plants. It was noticed that Chlorophyll a and chlorophyll b were higher in 1 mM JA treatment in comparison to 0.1 mM JA. In comparison to aphid-stressed plants, control plants had higher chlorophyll content. It was noted that aphid infestation lowered the carotenoid content in wheat plants, and JA treatment increased this content in both varieties. (Fig. 2).

### Antioxidants (SOD, POD and CAT)

SOD, POD, and CAT activities varied among the treatments. These activities were significantly lower during aphid stress compared to control. In contrast, non-sprayed plants, aphid-infested plants that received JA spray, had considerably increased SOD, POD, and CAT levels. In aphid-stressed plants, SOD activity was observed to be slightly lower (3.47 ± 0.4 EU/mg Protein), which was enhanced after JA applications (13.26 ± 2.7 to 14.18 ± 2.6 EU/mg Protein). POD and CAT activities in aphid stress plants were noticed at 2.93 ± 1.3 and 0.85 ± 0.3 EU/mg Protein in Borlaug-2015. The greater content of SOD, POD and CAT was observed in 1 mM JA sprayed plants compared to the 0.1 mM sprayed plants. Though control plants had higher activities of SOD, POD, and CAT (6.105 ± 0.1, 4.25 ± 1.5, 1.24 ± 0.7 EU/mg Protein) compared to those infested by aphids after JA treatment, this increase was higher in aphid infested plants compared to control plants (Table 1). In Zincol-2015 aphid-stressed plants, JA enhanced SOD activity from 5.74 ± 4.5 to 15.67 ± 3.5 EU/mg Protein, POD activity from 1.816 ± 2 to 7.101 ± 3 EU/mg Protein, and CAT activity from 0.01 ± 0.9b to 2.522 ± 1.9 EU/mg Protein (Table 2).

**Table 1:** Impact of foliar jasmonic acid treatment on SOD, CAT and POD content. Values in the table are just one variety Borlaug-2015. Values are mean ± SE (*n* = 3). Different letters within a column indicate significant difference between the treatments.

Treatments	SOD (EU/mg Protein)	POD (EU/mg Protein)	CAT (EU/mg Protein)
Control	6.105 ± 0.1^ab^	4.25 ± 1.5^a^	1.24 ± 0.7^a^
0.1 mM JA	7.57 ± 0.5a^b^	4.91 ± 1.7^a^	1.16 ± 0.3^a^
1 Mm JA	8.67 ± 2.8^ab^	5.45 ± 2.6^b^	1.82 ± 1^a^
Aphid	3.47 ± 0.4^b^	2.93 ± 1.3^a^	0.85 ± 0.3^b^
Aphid+0.1 mM JA	13.26 ± 2.7^ab^	7.75 ± 3.9^a^	2.51 ± 1.2^ab^
Aphid+1 Mm JA	14.18 ± 2.6^a^	8.65 ± 2.1^a^	2.69 ± 1.3^ab^

**Table 2:** Impact of foliar jasmonic acid treatment on SOD, POD and CAT content. Values in the table are variety Zincol-2015. Values are mean ± SE (*n* = 3). Different letters within a column indicate significant difference between the treatments (*p* < 0.05).

Treatments	SOD (EU/mg Protein)	POD (EU/mg Protein)	CAT (EU/mg Protein)
Control	7.438 ± 3.1^ab^	2.947 ± 2.4^a^	2.115 ± 0.8^a^
0.1 mM JA	8.01 ± 2.4^ab^	4.414 ± 2.2^a^	1.498 ± 0.4^a^
1 Mm JA	9.74 ± 6.2^ab^	9.429 ± 1.4^a^	1.248 ± 1.3^a^
Aphid	5.74 ± 4.5^b^	1.816 ± 2^b^	0.01 ± 0.9^b^
Aphid+0.1 mM JA	14.57 ± 4.2^a^	9.836 ± 1.8^a^	4.42 ± 1.4^a^
Aphid+1 Mm JA	15.67 ± 3.5^a^	7.101 ± 3^a^	2.522 ± 1.9^a^

### Total soluble protein, phenolics, MDA and H_2_O_2_

**Figure 3:**
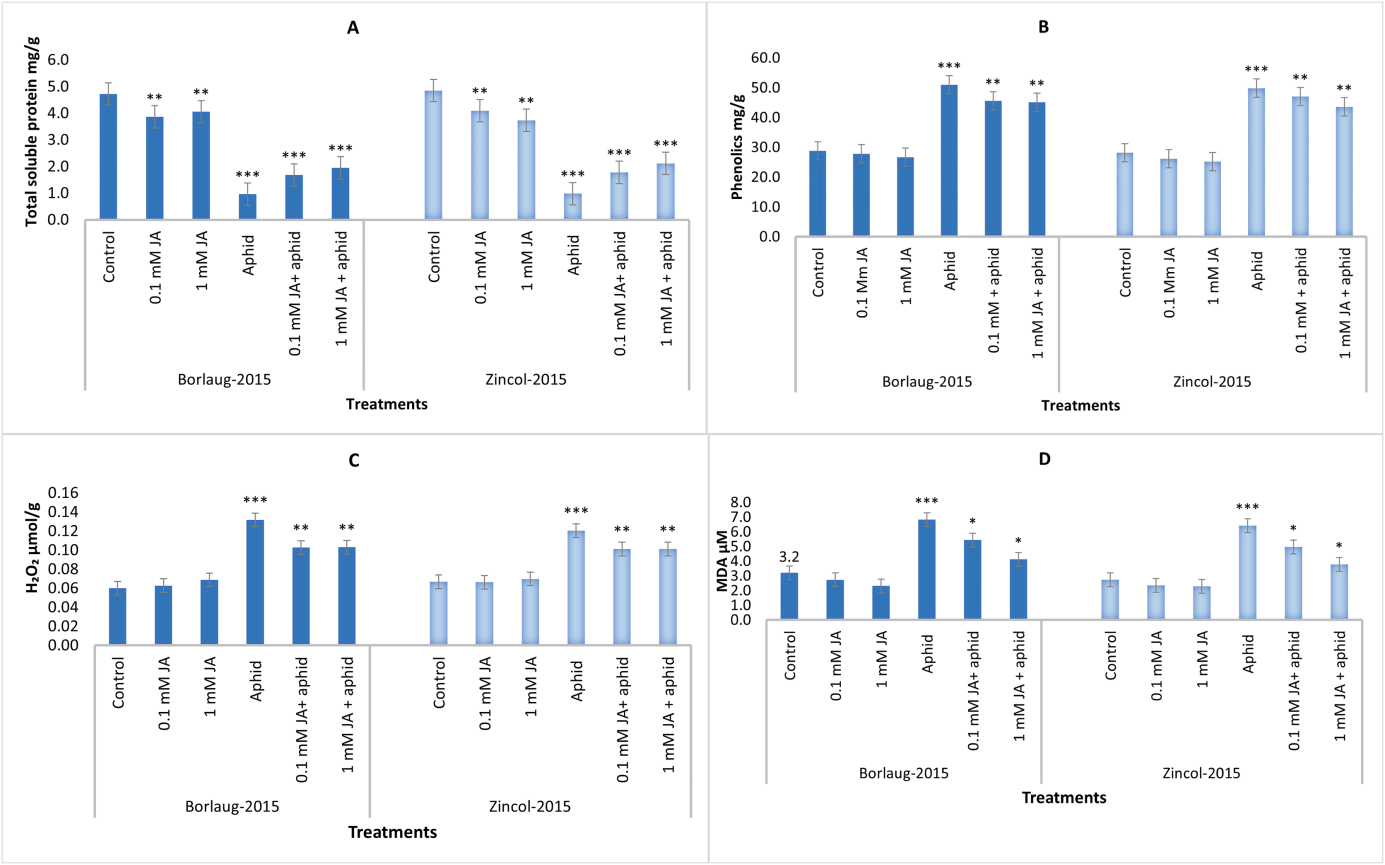
Impact of foliar jasmonic acid treatment on Total soluble protein (A), phenolics (B), H_2_O_2_ (C) and MDA content (D) of two varieties of wheat *i.e*., Borlaug-2015 and Zincol-2015 with and without aphid stress. Values are per gram fresh weight of sample. Mean values ± SE of three replicates are presented. *p ≤ 0.05; **p ≤ 0.01; ***p ≤ 0.001.

Aphid-stressed plants had less TSP content (1.0 mg/g f.wt) in contrast to control plants in both varieties. After JA treatments, aphid-infested plants showed a significant rise in TSP. In aphid-infested plants, jasmonic acid spray improved the TSP content from 1.7 to 1.9 mg/g f.wt in Borlaug-2015 and 1.8 to 2.1 mg/g f.wt in Zincol-2015. The results indicated that aphid-stressed plants had a greater content of phenolics as compared to control plants that declined after the application of JA. Lower phenolic content was observed in 1 mM JA sprayed plants compared to the 0.1 mM sprayed plants. In both wheat varieties, aphid stress led to a considerable rise in H_2_O_2_ and MDA concentration. Control plants had less H_2_O_2_ (0.06 µmol/g f.wt) and MDA (3.2 and 2.7 µmol/g f.wt) in comparison to the aphid-stressed plants (6.8, 6.4 MDA µmol/g f.wt and 0.13, 0.12 H_2_O_2_ µmol/g f.wt). Plants that received higher jasmonic acid 1 mM possessed low MDA and H_2_O_2_ (Fig. 3).

### Direct count of aphids

A population of aphids was counted prior to jasmonic acid spray and after 1, 3, and 7 days. Results indicated that 1 day after JA application, the aphid population declined. Aphids population significantly lowered after JA application in both varieties. Plants sprayed with a higher concentration of JA (1 mM) had fewer aphids. Plants that were not treated with JA bear the highest aphid population. The aphid population recorded before JA treatment was 49 ± 6 in Borlaug-2015 and 38 ± 2 in Zincol-2015. The aphid population declined after 1 day of JA treatment. Both varieties in 0.1 the mM JA treatment had 11 ± 4 and 12 ± 3 numbers of aphids, respectively, while 1 mM had JA 9 ± 8 and 4 ± 3. After 7 days, the population of aphids again increased in both wheat varieties, with a higher count in the 0.1 mM JA treatment than the 1 mM JA. Zincol-2015 showed a relatively lower number of aphids than Borlaug-2015 (Table 3).

**Table 3:** Comparative effect of different levels (0.1 mM, 1 Mm) of JA foliar application on aphid population in two wheat varieties, Borlaug-2015 and Zincol-2015, at different time intervals. Mean ± SE (*n* = 3). Different letters within a column indicate significant difference between the treatments (*p* < 0.05).

Time interval	Treatments	Borlaug-2015	Zincol-2015
Before JA spray	Aphid	49 ± 6^a^	38 ± 2^a^
	Aphid+0.1 mM JA	25 ± 8^b^	23 ± 7^b^
	Aphid+1 mM JA	41 ± 5^a^	25 ± 6^b^
After 1 day of spray	Aphid	49 ± 2^a^	40 ± 4^a^
	Aphid+0.1 mM JA	11 ± 4^b^	12 ± 3^b^
	Aphid+1 mM JA	9 ± 8^c^	4 ± 3^c^
After 3 days	Aphid	54 ± 9^a^	46 ± 2^a^
	Aphid+0.1 mM JA	16 ± 4^b^	14 ± 5^b^
	Aphid+1 mM JA	15 ± 2^c^	7 ± 1^c^
After 7 days	Aphid	58 ± 2^a^	46 ± 5^a^
	Aphid+0.1 mM JA	15 ± 6^b^	11 ± 1^b^
	Aphid+1 mM JA	14 ± 5^c^	9 ± 2^c^

## Discussion

It is generally recognized that aphid infestation has an adverse influence on wheat development and yield. This results in reduced plant height and other yield-related morphological attributes of plants (Kalaisekar, Padmaja & Patil, 2019; Wang et al., 2020). Foliar application of JA enhanced the plant growth in wheat as it increased the plant height, length of roots and shoots (Anjum et al., 2016). Similar findings showed increased plant growth in soybean (Anjum et al., 2011) and maize (Abdelgawad, Khalafaallah & Abdallah, 2014). In addition to length improvements, JA also boosted the fresh weight of wheat shoots and roots (Ilyas et al., 2017; Ali et al., 2020). Exogenous application of JA can promote plant growth in addition to pest resistance (Bhavanam & Stout, 2021). In wheat, it can reduce the damage caused by thrips and midges (Mouden et al., 2017).

In the current study, the experiment was conducted on two wheat varieties (Borlaug-2015 and Zincol-2015) to evaluate the influence of jasmonic acid spray against aphid population and its effect on the growth of plants. Plant biomass and photosynthetic ability and pattern are important plant traits that ultimately affect plants’ yield. We observed that plant biomass attributes as root/shoot length and plant weight along with leaf relative water content decreased as aphid stress increased. Similar observations are reported in safflower (Ghassemi-Golezani & Hosseinzadeh-Mahootchi, 2015), cotton (Abeed & Dawood, 2020) and wheat (Abeed, Eissa & Abdel-Wahab, 2021). In different plant species, the overall effect on leaf water content was similar: a foliar spray of JA significantly impacted and resulted in increased relative leaf water content (Nouri-Ganbalani et al., 2018).

In current work, aphid infested plants had lower Chlorophyll a, chlorophyll b and total chlorophyll content than the control. Other studies also showed that aphid infestation declines the chlorophyll content in wheat (Shahzad et al., 2019), and also in sorghum (Najali et al., 2018). Externally applied jasmonic acid raised the photosynthetic pigments in our experiment, which is consistent with previous reports (Ali et al., 2020; Abbaspour & Rezaei, 2014; Siddiqi & Husen, 2019). Under biotic or abiotic stress, plants have a very organized system to scavenge ROS generated after stress (Berner & Van der Westhuizen, 2010; Mansoor et al., 2022). These enzymatic/non-enzymatic scavengers (antixoidants) are generated in various tissues to minimize the effect of ROS by various signaling cascades (Kerchev & Van Breusegem, 2022). Aphid stress led to lower levels of antioxidant enzymes in our study. JA significantly protected the wheat from aphid stress conditions by magnifying the defense activities with enhanced activity of antioxidant enzymes. It raised SOD, CAT, and POD production and activity that effectively protect plants from a stress injury. It is also reported that POD and SOD levels in aphid-resistant wheat are greater than that in aphid susceptible wheat after the aphid infestation (Qi et al., 2020). Enzymes like superoxide dismutase (SOD) and catalase (CAT) can instantly detoxify hydrogen peroxide and other ROS at the site of production (Czerniewicz et al., 2017). Similar observations were obtained in the current experiment that Zincol-2015 (which appeared tolerant against aphid attack) had higher levels of both enzymes after JA spray in aphid-infested plants.

In the present study, aphid-stressed plants had a higher content of H_2_O_2_ and MDA as compared to the control. Increased H_2_O_2_ after aphid stress indicates that ROS signaling pathway is activated in response to aphid stress (Qi et al., 2020). Treatment of JA lowered the concentration of MDA, as well as H_2_O_2_ level in stressed plants (Abeed, Eissa & Abdel-Wahab, 2021). Hence, resulted in a lower accumulation of ROS during stress and improved the level of osmoprotectants (Kim et al., 2021). We also observed lower TSP in aphid stress plants compared to control plants. A significant rise in TSP was observed in aphid-infested plants after JA spray and was consistent with the results of previous studies (Wang et al., 2020; Abeed, Eissa & Abdel-Wahab, 2021). Moreover, increased phenolic content during the aphid infestation was observed and decreased after JA treatment. In previous reports, a similar pattern was obtained (Kaur, Salh & Singh, 2017; Złotek et al., 2019).

Moreover, the plants treated with JA had a lower population of aphids than untreated plants. Those plants sprayed with higher concentration of JA possessed lower aphid number compared to the lower level of JA. Previous studies showed that JA treatment could promote resistance mechanisms against insect herbivores and produce aphid resistance in wheat plants (Bayram & Tonğa, 2018; El-Wakeil & Volkmar, 2012; El-Wakeil, Volkmar & Sallam, 2010). There was a noticeable variation in the number of aphids among JA treatments in both varieties. Moreover, varietal differences were also observed as Borlaug-2015 appeared susceptible compared to Zincol-2015 against aphid attack.

## Conclusion

Results revealed that morphological attributes decreased under aphid stress and were improved after JA treatment. Photosynthetic pigments decreased under stress, whereas JA application helped the plants to recover these pigments. The activities of SOD, CAT, and POD in wheat were significantly reduced by aphid stress compared to the control. The antioxidant system was activated by JA, which enhanced the ROS scavengers’ levels. Compared to control plants, aphid-stressed plants had higher MDA and H_2_O_2_ concentrations. The results revealed that the aphid population is lower in JA sprayed plants than untreated plants. JA higher concentration (1 mM) was more effective than 0.1 mM to recover from aphid stress. Zincol-2015 appeared tolerant against aphid attacks as compared to Borlaug-2015. Consequently, in addition to adopting resistant varieties, farmers might integrate jasmonic acid treatments for crop protection, as JA would aid in developing ecologically friendly crops by reducing the use of insecticides/pesticides. It is worthy to point out that a lower pest population is not always complemented by enhanced plant growth. Hence, further validations are needed for field applications and to devise a proper mode of JA application for pest control and enhanced plant yield.

## Supplemental Information

10.7717/peerj.14018/supp-1 Supplemental Information 1 Raw data for morphological parameters.

10.7717/peerj.14018/supp-2 Supplemental Information 2 Raw data for Chlorophyll.

10.7717/peerj.14018/supp-3 Supplemental Information 3 Raw data for SOD.

10.7717/peerj.14018/supp-4 Supplemental Information 4 Raw data for MDA and H2o2.

10.7717/peerj.14018/supp-5 Supplemental Information 5 Raw data for POD.

10.7717/peerj.14018/supp-6 Supplemental Information 6 Raw data for CAT.

10.7717/peerj.14018/supp-7 Supplemental Information 7 Raw data for TSP.

## Additional Information and Declarations

### Data Availability

The following information was supplied regarding data availability: The raw data is available in the Supplemental Files.
